# Oral health-related quality of life and pain perception among adult patients treated with clear aligners: a longitudinal prospective study

**DOI:** 10.1590/2177-6709.30.2.e2524169.oar

**Published:** 2025-05-23

**Authors:** Sonia Patricia PLAZA-RUÍZ, Judith Patricia BARRERA-CHAPARRO, Alejandra ECHEVERRÍA-ZARAMA, Melissa Paola ROJAS-ROMÁN, Keila Stefania SUSA-VALENCIA

**Affiliations:** 1 University Foundation CIEO-UniCIEO, Dental School, Department of Orthodontics (Bogotá, Colombia).

**Keywords:** Orthodontics, corrective, Pain perception, Quality of life, Oral health, Orthodontic appliances, removable, Ortodontia corretiva, Percepção da dor, Qualidade de vida, Saúde bucal, Aparelhos ortodônticos removíveis

## Abstract

**Objective:**

To compare the impact on Oral Health Related Quality of Life (OHRQoL) and pain perception between adult patients undergoing fixed conventional bracket treatment (FCBT) and those undergoing clear aligner treatment (CAT) during the first three months of orthodontic treatment (OT).

**Method:**

OHRQoL was measured by the Oral Health Impact Profile (OHIP-S14 Ortho), pain perception was evaluated by the visual analog scale (VAS). The on-line questionnaires were administered immediately before OT (T0), 24-48 hours (T1), one month (T2), and three months (T3) after appliance installation. Group 1 (n = 48; 31.21 ± 11.47 years) was comprised of patients with FCBT from an orthodontic program. Group 2 (n=44; 38.30 ± 11.91 years) was comprised of patients with CAT from a private office. Chi-squared, Mann-Whitney U and Friedman tests were used for analysis.

**Results:**

In the comparison between the groups, a statistically significant difference was observed at T0, with the CAT group showing higher OHIP-S14 Ortho scores than the FCBT group (P=0.0237). However, no significant differences were found at T1, T2, or T3. OHIP-S14 Ortho scores in the FCBT group increased significantly (P<0.05) at T1-T0 and T3-T0, while CAT scores decreased. However, this trend reversed at T2-T1. Pain perceptions were lower in the CAT group at T0, T1, and T3 and at T2-T1 (P<0.01).

**Conclusions:**

In the early stages of OT, the CAT group demonstrated improvements in QoL compared to baseline, while the FCBT group showed a decrease. The negative impact on OHRQoL and pain perception over the three months of follow-up tended to decrease after 24-48 hours in both the FCBT and CAT groups. Pain perception was more negatively affected in FCBT than in the CAT group.

## RESUMO

**Objetivo:**

Comparar o impacto na Qualidade de Vida Relacionada à Saúde Bucal (QVRSB) e a percepção da dor entre pacientes adultos submetidos ao tratamento convencional com braquetes fixos (BF) e aqueles submetidos ao tratamento com alinhadores transparentes (AT) durante os três primeiros meses do tratamento ortodôntico (TOrtod).

**Métodos:**

A QVRSB foi medida pelo *Oral Health Impact Profile* (OHIP-S14 Ortho) e a percepção da dor foi avaliada pela escala visual analógica (EVA). Os questionários on-line foram aplicados imediatamente antes do TOrtod (T0), 24-48 horas (T1), um mês (T2) e três meses (T3) após a instalação do aparelho. O Grupo 1 (n = 48; 31,21 ± 11,47 anos) foi composto por pacientes com BF de um programa ortodôntico. O Grupo 2 (n = 44; 38,30 ± 11,91 anos) foi composto por pacientes com AT de um consultório particular. Os testes qui-quadrado, U de Mann-Whitney e de Friedman foram usados para análise.

**Resultados:**

Na comparação entre os grupos, foi observada uma diferença estatisticamente significativa em T0, com o grupo AT apresentando escores OHIP-S14 Ortho mais altos do que o grupo BF (P=0,0237). Entretanto, não foram encontradas diferenças significativas em T1, T2 ou T3. Os escores do OHIP-S14 Ortho no grupo BF aumentaram significativamente (P<0,05) em T1-T0 e T3-T0, enquanto os escores do AT diminuíram. No entanto, essa tendência se inverteu em T2-T1. As percepções de dor foram menores no grupo AT em T0, T1 e T3 e em T2-T1 (P<0,01).

**Conclusões:**

Nos estágios iniciais do TOrtod, o grupo AT demonstrou melhorias na QVRSB, em comparação com o *baseline*; enquanto o grupo BF apresentou uma diminuição. O impacto negativo na QVRSB e na percepção da dor durante os três meses de acompanhamento tendeu a diminuir após 24-48 horas nos grupos BF e AT. A percepção da dor foi mais negativamente afetada no grupo BF do que no grupo AT.

## INTRODUCTION

Malocclusion can lead to functional limitations, dissatisfaction with the dentofacial appearance, and negatively impact oral health-related quality of life (OHRQoL).^1^ ^-^ ^3^ Orthodontic treatment (OT) aims to correct malocclusion; however, it may also have negative effects on the patient’s daily life, such as pain, difficulty in eating or smiling, and functional limitations.^4^ Several authors^5^ ^-^ ^8^ have found that the most significant decrease in OHRQoL occurs during the early phase of OT, causing difficulties in chewing, discomfort, pain, and functional limitations, with the most pronounced effects occurring 24-48 hours after brackets placement. 

Over the years, different devices have been developed for OT, such as fixed conventional brackets treatment (FCBT), self-ligating brackets, and clear aligner treatment (CAT) - which consists of removable transparent polyurethane splints (aligners) manufactured using computer-aided design (CAD) technology. Fixed appliances are the standard treatment for adolescents and adults; however, many adults now prefer more aesthetic options, leading to a growing demand for CAT.^9^ Patients often inquire about differences in comfort and the impact on quality of life (QoL). 

Although the impact of different orthodontic appliances on OHRQoL has been previously studied,^4^ ^,^ ^10^ the literature presents contradictory results. Shalish et al.^4^ and AlSeraidi et al.^11^ found that patients undergoing CAT reported the highest overall QoL scores. Gao et al.^12^ observed that patients treated with CAT experienced lower pain levels, less anxiety, and higher OHRQoL, compared to those treated with fixed appliances. Alajmi et al.^13^ reported that, while CAT affected pronunciation and speech delivery in the short term, it was more tolerable due to better patient satisfaction with food consumption and lower likelihood of causing mucosal ulcerations, compared to FCBT. Flores-Mir et al.^14^ found that FCBT and CAT had statistically similar satisfaction outcomes, except in eating and chewing, where the CAT group reported greater satisfaction. Zhang et al.^15^ conducted a systematic review that found weak evidence suggesting that CAT is less likely to cause eating disturbance than FCBT. However, many of these studies suffer from methodological biases, including small sample sizes, the use of non-validated questionnaires, assessments conducted only at the end of treatment, and reliance on subjective measures of patient satisfaction. These issues can introduce bias and limit the reliability of the findings. 

Additionally, experiencing pain during OT can negatively impact patient’s QoL.^16^ Some studies have found that patients treated with CAT report lower levels of pain, compared to those treated with FCBT, during the initial phases of OT.^17^ ^,^ ^18^

Thus, the aim of the present study was to compare the impact on OHRQoL and pain perception between adult patients undergoing FCBT and those undergoing CAT during the first three months of OT.

## MATERIAL AND METHODS

In this longitudinal prospective study, the STROBE guidelines for observational studies were followed.^19^ The sample consisted of patients who met the selection criteria and voluntarily agreed to participate. These patients were consecutively assigned to two groups, based on the orthodontic appliance used: Group 1 consisted of 48 patients with FCBT (Gemini 3M Unitek Orthodontic Products, CA, USA), who attended the orthodontic program clinic of the Fundación Universitaria CIEO - UniCIEO (UniCIEO); Group 2 consisted of 44 patients with CAT (Invisalign^®^; Align Technology, Santa Clara, CA, USA), who attended a private clinic in Bogotá, Colombia. 

The study was conducted from October 2021 to September 2023. The protocol was approved by the research ethics committee of UniCIEO (ethical endorsement #148, certificate 70). All participants voluntarily agreed to participate in the study and signed an informed consent form. 

The sample size was calculated based on the OHIP-S14 score. A mean difference of 5, with standard deviations of 9 in the FCBT group and 6 in the CAT group, was assumed, according to data from a previous study,^20^ with a power of 0.80 and a confidence level of 95%. As a result, 38 patients were required in each group. 

The inclusion criteria were patients aged over 18 years who were beginning treatment with CAT or FCBT and had dental aesthetic index (DAI) scores of 26-30 (definitive malocclusion/elective OT).^21^ ^,^ ^22^ Patients with a history of systemic disease, those requiring surgical treatment, those with cognitive disorders, or those who did not understand Spanish were excluded. 

The primary outcome measure was OHRQoL, assessed using the OHIP-S14 Ortho instrument, which was derived from the original OHIP-49 and specifically validated in Spanish for a population of patients undergoing orthodontic treatment by Barrera-Chaparro et al.^23^ The scale consists of 14 questions, two for each following dimension: functional limitation, physical pain, psychological discomfort, physical disability, psychological disability, social disability, and handicap. A Likert-type scale was used for quantification (0=never, 1= almost never, 2= sometimes, 3= frequently, 4= almost always). The score was calculated to obtain values between 0 and 56, with the higher scores indicating a greater negative impact on OHRQoL. The secondary outcome was pain perception, measured using a visual analog scale (VAS) from 0 to 10, where 0 represents no pain and 10 represents the worst pain imaginable_._ ^24^

The OHIP-S14 Ortho and pain questionnaires were self-administered: the OHIP questionnaire was completed four times, and the VAS, three times during the first three months of OT. Data were collected at T0 (immediately before appliance placement), T1 (24-48 hours after placement), T2 (one month after placement), and T3 (three months after placement). 

The parameters of the Checklist for Reporting Results of Internet E-Surveys (CHERRIES) were followed. ^25^ The online survey was hosted on SurveyMonkey^®^ (Momentive Inc., San Mateo, CA, USA) and distributed via WhatsApp application (WhatsApp. Inc., Mountain View, CA, USA). Additionally, sociodemographic variables, such as sex, age, educational level, and socioeconomic stratification^26^ were collected at T0.

## STATISTICAL ANALYSIS

Statistical analysis was performed using STATA 16 software (version 16, StataCorp, College Station, TX, USA). The statistician was blinded during the analysis, coding the groups with letters instead of using the treatment names. To establish bivariate associations, chi-squared tests were performed for qualitative variables, and the Mann-Whitney U-test was applied for quantitative variables, due to the non-normal distribution of the data. To evaluate the association between OHRQoL and pain perception with the treatment types over time, Friedman tests were used. Statistical significance was considered at p<0.05. 

## RESULTS

**Figure 1:**
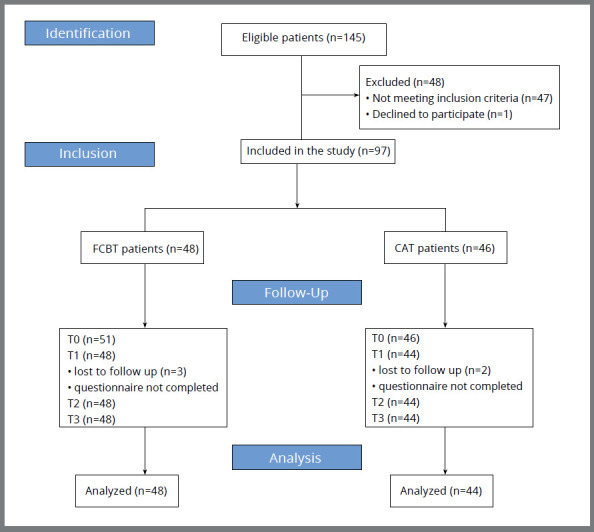
STROBE Flow diagram.

A total of 149 patients entered the study, of whom 48 did not meet the inclusion criteria. Five patients were excluded for not completing the survey on time at T1. Thus, the final sample comprised 92 patients (48 in FCBT group and 44 in CAT group) (Fig. 1, Table 1). 

**Table 1:** Distribution of variables in groups at baseline (T0).

Variable	Bracket system						P value
	FCBT (n=48)			CAT (n=44)			
Qualitative variables	n (%)			n (%)			
School level							
Secondary	21 (43.75)			1 (2.27)			<0.0001Ŧ****
Technician	0 (0.00)			1 (2.27)			
University	27 (56.25)			38 (86.36)			
Postgraduate	0 (0.00)			4 (9.09)			
Occupation							
Student	9 (18.8)			1 (2.3)			<0.023†*
Employee	27 (56,3)			33 (75.0)			
Independent	11 (22,9)			6 (13.6)			
Informal worker	0 (0.0)			3 (6.8)			
Retired	1 (2.1)			1 (2.3)			
Socioeconomic strata							
Strata 2	13 (27.08)			0 (0.00)			<0.0001Ŧ****
Strata 3	19 (39.58)			8 (18.18)			
Strata 4	12 (25.00)			17 (38.64)			
Strata 5	4 (8.33)			9 (20.45)			
Strata 6	0 (0.00)			10 (22.73)			
Sex							
Female	27 (56.25)			31 (70.45)			0.159 Δ
Male	21 (43.75)			13 (29.55)			
Quantitative variables	Median	Min-max	Mean (SD)	Median	Min-max	Mean (SD)	
Age	29	18-64	31.21 (11.47)	35	19-69	38.30 (11.91)	0.0013Ψ**
DAI	27	26-30	27.35 (1.158)	27	26-30	27.39 (1.02)	0.8880¶
OHIP-S14 Ortho	18.5	0-48	20.22 (11.15)	23	14-49	25.02 (8.52)	0.0237 Ψ**

FCBT: fixed conventional bracket treatment, CAT: clear aligners treatment, OHIP-S14 Ortho: Oral Health Impact Profile 14 Ortho. Statistically significant at *P <0.05, **** P <0.0001. † Chi^2^ test. Ŧ Exact Fisher test. ψ U Mann-Whitney test. ¶ t test.

Statistically significant differences (p<0.05) were found between the groups regarding educational level, socioeconomic status, age, occupation, and the OHIP-S14 Ortho. 

**Figure 2:**
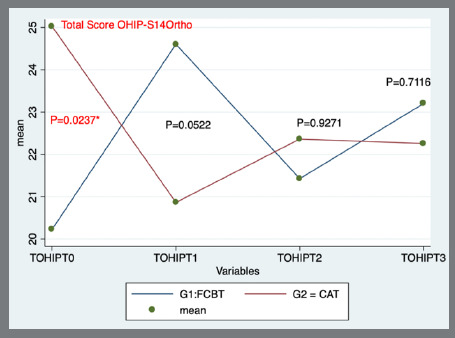
Total score OHIP-S14 Ortho.

Comparison of the median OHIP-S14 Ortho total scores between the groups at follow-up time indicated statistically significant differences (p=0.0237) at T0, with a higher score for the CAT group. At T1, T2, and T3, the score was higher for the FCBT group (Table 2, Fig. 2).

**Table 2:** Association between oral health quality of life (OHIP-S14 Ortho scores) and orthodontic appliance treatment, across time intervals (T0, T1, T2, and T3).

Variable	Bracket system						P value
OHIP-S14 Ortho (total score)	FCBT (n=48)			CAT (n=44)			
	Median (IQR)	Min	Max	Median (IQR)	Min	Max	
T0	18.5 (14)	16.99	23.47	23 (11)	22.43	27.61	0.0237 *¶
T1	23.5 (18)	21.67	27.54	20 (5.5)	18.92	22.81	0.0522 ψ
T2	22 (14)	18.73	24.12	22 (9)	20.01	24.72	0.9271 ψ
T3	22 (15.5)	19.54	26.86	19 (9)	19.56	24.95	0.7116 ψ

FCBT: fixed conventional bracket treatment, CAT: Clear aligner treatment. T0: Before orthodontic appliance placement, T1: 24-48 hours after orthodontic appliance placement, T2: one month after orthodontic appliance placement, T3: three months after orthodontic appliance placement. IQR = Interquartile Range. Statistically significant at *P <0.05 with Bonferroni correction for multiple tests. ψ U Mann-Whitney test. ¶ t Student test.

Statistically significant differences (p<0.05) in changes in OHRQoL between the groups during the follow-up time were found at T1-T0, T3-T0, and T2-T1. At T1-T0 and T3-T0, the median of the OHIP-S14 Ortho total score decreased for the CAT group, while it increased for the FCBT group. In contrast, at T2-T1, the median of the OHIP-S14 Ortho total score increased for the CAT group, while it decreased for the FCBT group (Table 3). 

**Table 3:** Relative changes in oral health quality of life (OHIP-S14 Ortho scores) across time intervals according to the orthodontic appliance system.

Variable	Orthodontic appliance system						P value
OHIP-S14 Ortho (Total score)	FCBT (n=48)			CAT (n=44)			
	Median (IQR)	Min	Max	Median (IQR)	Min	Max	
T1 - T0	3.5 (9.5)	-18	24	-4 (-8.5)	-28	21	0.0001****
T2 - T0	1 (-15.0)	-25	28	-4 (-8.0)	-27	27	0.0979
T3 - T0	2.5 (-15.0)	-19	31	-4 (-11.0)	-32	31	0.0389*
T2 - T1	-3 (-11.0)	-29	24	1(-4.0)	-10	16	0.0026**
T3 - T1	-2.5 (-11.5)	-23	32	0 (0.0)	-11	19	0.059
T3 - T2	-2 (3.0)	-28	15	0 (-5.0)	-23	9	0.0527

FCBT: Fixed conventional bracket treatment, CAT: Clear aligners treatment. T0: Before orthodontic appliance placement, T1: 24-48 hours after orthodontic appliance placement, T2: one month after orthodontic appliance placement, T3: three months after orthodontic appliance placement. IQR = Interquartile Range. Statistically significant at *P <0.05, **P <0.01, ****P <0.0001, with Bonferroni correction for multiple tests. U-Mann Whitney test.

Comparison of the changes in OHIP-S14 Ortho scores for the seven domains between groups indicated statistically significant differences (p<0.05) for functional limitations, physical pain, psychological discomfort, physical disability and psychological disability (Table 4). 

**Table 4:** Comparison of medians of overall and domain scores in OHRQoL across time intervals.

Variable	T1-T0	P	T2-T0			P	T3-T0			P	T2-T1			P	T2-T3			P
	Median Min/ Max		Median Min/ Max				Median Min/ Max				Median Min/ Max				Median Min/ Max			
OHIP-S14 Ortho Total Score		0.0001****				0.0979				0.0389**				0.0026**				0.0527
FCBT	3.5 -18 / 24		1 -25 / 28				2.5 -19 / 31				-3 -29 / 24				-2 -28 / 15			
CAT	-4 -28 / 21		-4 -27 / 27				-4 -32 / 31				1 -10 / 16				0 -23 / 9			
Functional Limitation		0.0000****				0.0743				0.0677				0.0025**				0.7271
FCBT	1 -4 / 6		0 -5 / 5				0 -5 / 8				-1 -4 / 3				0 -4 / 4			
CAT	-1 -4 / 3		-1 -4 / 3				-1 -5 / 6				0 -3 / 3				0 -4 / 3			
Physical pain		0.0001****				0.3896				0.0053***				0.0000****				0.0100**
FCBT	2 -2 / 8		0 -7 / 6				2 -2 / 8				-2 -8 / 3				-1 -6 / 4			
CAT	0.5 -4 / 7		4 -4 / 5				0 -7 / 6				0 -3 / 3				0 -4 / 3			
Psychological discomfort		0.0000****				0.0613				0.0408*				0.0033**				0.2671
FCBT	0 -4 / 5		0 -6 / 4				0 -5 / 6				0 -2 / 3				0 -5 / 3			
CAT	-2 -7 / 3		-1 -8 / 4				-1 -8 / 4				0 -6 / 4				0 -4 / 2			
Physical disability		0.0029**				0.0539				0.0594				0.0692				0.5634
FCBT	1 -4 / 5		0 -4 / 6				0.5 -4 / 5				-1 -4 / 6				0 -5 / 2			
CAT	0 -18 / 4		-0.5 -17 / 4				0 -16 / 5				0 -3 / 2				0 -4 / 3			
Psychological disability		0.0093**				0.2463				0.0811				0.0972				0.1255
FCBT	0 -5 / 3		0 -7 / 4				0 -4 / 5				0 -8 / 2				-0.5 -4 / 4			
CAT	0 -7 / 3		0 -7 / 4				0 -7 / 5				0 -4 / 4				0 -3 / 3			
Social disability		0.7218				0.9411				0.8249				0.7695				0.1849
FCBT	0 -7 / 3		0 -4 / 6				0 -3 / 3				0 -4 / 8				0 -5 / 2			
CAT	0 -5 / 3		0 -5 / 4				0 -5 / 4				0 -2 / 4				0 -4 / 1			
Handicap		0.9333				0.9166				0.6552				0.9466				0.1073
FCBT	0 -4 / 2		0 -4 / 3				0 -3 / 4				0 -2 / 6				0 -6 / 1			
CAT	0 -4 / 4		0 -5 / 4				0 -5 / 4				0 -2 / 4				0 -4 / 2			

FCBT: fixed conventional bracket treatment, CAT: clear aligners treatment. T0: Before orthodontic appliance placement, T1: 24-48 hours after orthodontic appliance placement, T2: one month after orthodontic appliance placement, T3: three months after orthodontic appliance placement. Statistically significant at *P <0.05, **P <0.01, *** P <0.001, **** P <0.0001 with Bonferroni correction for multiple tests. U-Mann Whitney test.

The perceptions of pain in the FCBT group were greater than those in the CAT group at all times; moreover, they decreased over time in the FCBT group, but increased at T2 and decreased at T3 in the CAT group (p<0.01) (Table 5). 

**Table 5:** Association between VAS and the bracket system across time intervals (T0, T1, T2, and T3).

Variable	Bracket system						P value
VAS (Total score)	FCBT (n=48)			CAT (n=44)			
	Median (IQR)	Min	Max	Median (IQR)	Min	Max	
T1	5.00 (2.50)	1.00	9.00	2.00 (3.00)	1.00	9.00	<0.0001****
T2	4.00 (3.00)	1.00	8.00	3.00 (2.00)	1.00	8.00	0.001 ***
T3	3.50 (2.00)	1.00	8.00	2.00 (2.00)	1.00	8.00	0.001 ***

FCBT: Fixed Conventional Bracket treatment, CAT: Clear aligners treatment. T1: 24-48 hours after orthodontic appliance placement, T2: one month after orthodontic appliance placement, T3: three months after orthodontic appliance placement. IQR = Interquartile Range. Statistically significant at **P <0.01, ***P <0.001, **** P <0.0001 with Bonferroni correction for multiple tests. U-Mann Whitney test.

Comparison of the VAS scores at the different time intervals (Table 6) indicated statistically significant differences (p=0.027) at T2-T1, with a greater decrease in VAS scores in the FCBT group. In the CAT group, the VAS score was lower than in the FCBT group, and remained almost constant during the time intervals. 

**Table 6:** Relative changes in VAS across time intervals according to the bracket system.

Variable	Bracket system						P value
VAS (Total score)	FCBT (n=48)			CAT (n=44)			
	Median (IQR)	Min	Max	Median (IQR)	Min	Max	
T2 - T1	-1.00 (-1.00)	-7.00	6.00	0.00 (0.00)	-7.00	3.00	0.027*
T3 - T1	-1.00 (-2.00)	-6.00	4.00	0.00 (0.00)	-7.00	3.00	0.069
T3 - T2	0.00 (0.00)	-5.00	4.00	0.00 (0.00)	-2.00	4.00	0.855

FCBT: fixed conventional bracket treatment, CAT: clear aligners treatment. T1: 24-48 hours after orthodontic appliance placement, T2: one month after orthodontic appliance placement, T3: three months after orthodontic appliance placement. IQR = Interquartile Range. Statistically significant at *P <0.05 with Bonferroni correction for multiple tests. U-Mann Whitney test.

**Figure 3:**
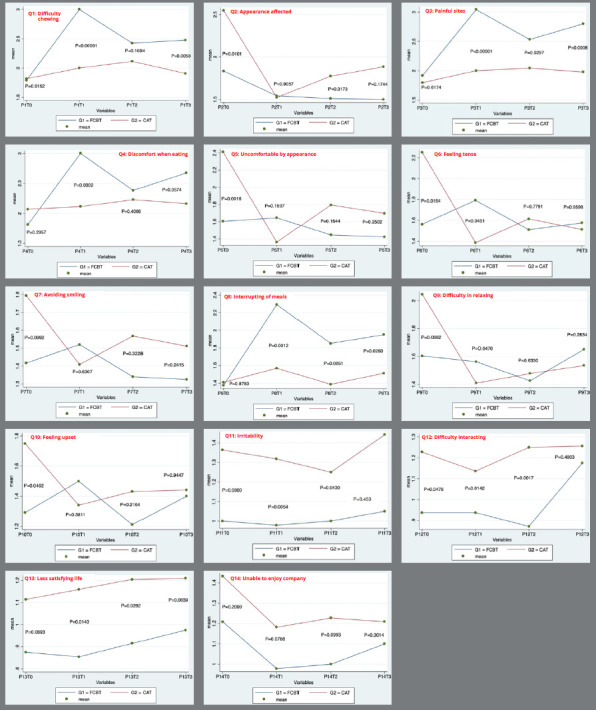
OHIP-S14 Ortho questions between groups.

The comparison of the 14 questions (Q) of the OHIP-S14 Ortho between the groups at various time intervals revealed that CAT patients had worse OHIP-S14 scores at baseline (T0) for Q2 (appearance affected), Q5 (uncomfortable with appearance), Q6 (feeling tense), Q7 (avoiding smiling), Q9 (difficulty in relaxing), and Q10 (feeling upset). In contrast, the FCBT group showed a higher negative impact on OHRQoL at T1 for Q1 (difficulty in chewing), Q4 (discomfort when eating), and Q6 (feeling tense), and throughout the study for Q3 (painful sites) and Q8 (interruption of meals). Additional details can be found in Figure 3. 

Friedman tests were conducted to determine whether intragroup OHIP-S14 Ortho scores and VAS scores differed over time; significant differences were found in both groups [x²(6), FCBT=179.33, p<0.00001; CAT=208.57, p<0.00001] for the OHIP-S14 Ortho scores, but not for the VAS scores [x²(2), FCBT=5.57, p=0.062; CAT=0.378, p=0.828]. 

## DISCUSSION

OT can enhance dentofacial aesthetics, occlusal functions, and patients’ QoL.^27^ ^-^ ^31^ While it is well-established that OHRQoL tends to decline during the first month of treatment^7^ ^,^ ^15^, the extension of the negative impact may vary depending on the type of appliance used.^32^

In the present study, the groups started (T0) with differences in OHRQoL, with the CAT group reporting worse QoL than the FCBT group. However, at T1, T2, and T3, the total OHIP scores were lower in the CAT group, compared to the FCBT group, though these differences were not statistically significant. Various studies^4^ ^,^ ^11^ ^,^ ^12^ have reported statistically significant improvements in OHRQoL over the time for CAT groups compared to FCBT groups. The findings in the present study may be influenced by the initial differences between the groups regarding sociodemographic variables and their impacts on OHRQoL. Notably, the CAT group exhibited a greater negative impact on QoL at T0, which could be attributed to a preference for this type of appliance among more anxious patients, as well as a tendency for highly educated individuals with greater financial resources to be more discerning patients.^33^ ^,^ ^34^

At T1 and T3, compared to T0, the CAT group showed improvements in OHRQoL, while the FCBT group experienced a decline. Conversely, at T2 compared to T1, the OHRQoL of the CAT group worsened, whereas it improved in the FCBT group. Gao et al.^12^ reported that patients using CAT experienced lower pain levels, reduced anxiety, and higher OHRQoL, compared to those receiving FCBT. Similarly, Jaber et al.^20^ found that overall OHIP-S14 scores significantly increased, peaking in both groups one week after treatment began; with OHRQoL being poorer in the FCBT group than in the CAT group. In the present study, it was observed that the overall OHIP-S14 score increased in the FCBT group, peaking 24-48 hours after bracket placement (T1).

This study found statistically significant differences between the groups in several domains of the OHIP-S14 scale, including functional limitation, physical pain, psychological discomfort, physical disability, and psychological disability. The FCBT group experienced a higher negative impact on OHRQoL, particularly in areas such as difficulty chewing, discomfort while eating, and feeling tense, painful sites and interruption of meals. Conversely, CAT patients reported worse OHIP-S14 scores related to appearance, discomfort with their appearance, feeling tense, avoiding smiling, difficulty in relaxing, feeling upset, irritability, difficulty interacting, less satisfying life and unable to enjoy company. These findings align with those of Flores-Mir et al.^14^, who noted that CAT patients reported greater satisfaction with eating and chewing, compared to FCBT patients. Similar conclusions were reported by Gao et al.^12^ and Alajmi et al.^13^, who suggested that, while treatment with aligners is not entirely pleasant, it may be more tolerable than FCBT, because it allows patients to meet their dietary needs and reduces the risk of mucosal ulceration. They also mentioned that clear aligners may affect pronunciation and speech in the short term. 

Marañón-Vásquez et al.^34^ found that patients who preferred CAT prioritized comfort and smile aesthetics when choosing an orthodontic appliance. Kuhlman et al.^35^ reported that adult patients with higher socioeconomic status viewed aesthetics as the most attractive attribute. In contrast, Shalish et al.^4^ found that the general activity and oral dysfunction issues of the group treated with aligners were comparable to those of patients undergoing FCBT. However, Knorst et al.^36^ found that individuals of lower socioeconomic status experienced worse OHRQoL. 

Most patients experience pain at some point during OT, and understanding pain patterns is crucial, as they significantly affect patients’ QoL.^17^ ^,^ ^18^ Krukenmeyer et al.^16^ suggested that fear of pain is one of the primary reasons for abandoning OT. This study found significant differences in pain perception between treatment groups, although intragroup differences over the three months of OT were not significant. Initially, the FCBT group reported higher levels of pain, compared to the CAT group, but this difference decreased over time. This pattern may be attributed to patients gradually adapting to the treatment and the fact that the most significant tooth movement occurs in the early phases of OT. Similar results have been reported by other authors.^11^ ^,^ ^12^ ^,^ ^37^ Moreover, other studies^17^ ^,^ ^18^ have observed higher pain levels in patients with fixed appliances during the first 24 hours of OT. In contrast, Shalish et al.^4^ found that Invisalign™ patients experienced relatively higher pain levels in the first few days after appliance placement, possibly due to the greater mechanical forces applied by aligners during the early stages of treatment. 

The results of this study have several clinical implications, particularly the observation that before starting OT, patients opting for CAT tend to be more demanding than those choosing FCBT. This difference may be attributed to their expectations for aesthetics and comfort, which are often influenced by their typically higher socioeconomic status.^38^ Patients willing to invest in more expensive treatments often expect better outcomes and smoother process.^39^ In contrast, FCBT patients may have more realistic expectations, based on the traditional orthodontic experience. Clinicians should manage the higher expectations of CAT patients, to ensure satisfaction throughout treatment. Another important finding of the present study for clinical practice is that pain perception associated with CAT was less than that experienced with FCBT. Lower pain levels can lead to increased patient comfort and satisfaction, and this difference in pain levels can influence treatment adherence, as patients experiencing less discomfort are more likely to follow treatment protocols.

One of the limitations of the present study was that the VAS scale was not applied at T0. It is important to have a baseline comparison value at the beginning of a study, as patients can often report some type of pain. Other limitations included differences between the groups at baseline regarding educational level, socioeconomic status, age, and OHIP-S14 scores. A potential limitation of the study is test-retest bias, as familiarity with the same questionnaire may influence responses. Additionally, important confounding factors, such as analgesic consumption and previous OT, were not included in the selection criteria. Furthermore, other confounding variables were not accounted for during the recruitment and data analysis phases. Although some confounding factors, such as sex and malocclusion severity, were controlled through selection criteria, the characteristics of patients recruited from different centers still varied. However, this scenario may mirror real-life situations where patients opting for higher-cost treatments have different expectations and perceptions, compared to those choosing most affordable options. Therefore, randomized controlled trials are recommended, as they can provide clearer results by randomizing patients to treatments, simulating an ideal scenario where the cost of treatment is not an influencing factor in group selection. 

The strengths of this study include the rigorous follow-up of participants during the first three months of treatment, allowing to assess their OHRQoL using a specific instrument for orthodontic patients, namely, the OHIP-S14Ortho^23^, as well as their pain levels. These findings offer valuable insights for clinicians, helping them manage patients’ expectations at the beginning of treatment.

However, the limitations discussed earlier should be considered, and the results should be interpreted with caution, and not generalized. Future research is recommended to compare CAT and FCBT until completion of OT, including patients with varying levels of malocclusion complexity, and to incorporate randomized and blinded group assignments, to strengthen the validity of the findings. 

## CONCLUSIONS

Although no statistically significant differences were found in OHIP-S14 scores ​​between the groups at T1, T2, and T3, the relative changes in QoL over time (T1-T0, T3-T0, and T2-T0) indicated a decrease in the CAT group and an increase in the FCBT group.

FCBT patients experienced more disturbances in chewing, eating, and feeling tension 24-48 hours after appliance placement, compared with CAT patients. On the other hand, CAT patients were more affected by concerns related to appearance and aesthetics, and exhibited more psychological difficulties prior to treatment. Additionally, the CAT group reported significantly lower pain perception during the first three months of OT, compared to the FCBT group.
